# Predicting early brain metastases based on clinicopathological factors and gene expression analysis in advanced HER2-positive breast cancer patients

**DOI:** 10.1007/s11060-014-1704-y

**Published:** 2015-01-06

**Authors:** Renata Duchnowska, Jacek Jassem, Chirayu Pankaj Goswami, Murat Dundar, Yesim Gökmen-Polar, Lang Li, Stephan Woditschka, Wojciech Biernat, Katarzyna Sosińska-Mielcarek, Bogumiła Czartoryska-Arłukowicz, Barbara Radecka, Zorica Tomasevic, Piotr Stępniak, Konrad Wojdan, George W. Sledge, Patricia S. Steeg, Sunil Badve

**Affiliations:** 1 Department of Oncology, Military Institute of Medicine, Szaserów St 128, 04-141 Warsaw, Poland; 2 Department of Oncology and Radiotherapy, Medical University, Gdańsk, Poland; 3 Center for Computational Biology and Bioinformatics, Department of Medical and Molecular Genetics, Indiana University School of Medicine, Indianapolis, IA USA; 4 Departments of Medicine and Pathology and Laboratory Medicine, Indiana University School of Medicine, Indianapolis, IA USA; 5 Women’s Cancers Section, Laboratory of Molecular Pharmacology, Center for Cancer Research, National Cancer Institute, Bethesda, MD USA; 6 Department of Pathology and Oncology, Medical University, Gdańsk, Poland; 7 Regional Oncology Center, Gdańsk, Poland; 8 Department of Oncology, Oncology Center, Białystok, Poland; 9 Department of Oncology, Oncology Center, Opole, Poland; 10 Institute for Oncology and Radiology, Belgrade, Serbia; 11 Transition Technologies S.A., Warsaw, Poland; 12 Institute of Heat Engineering, Warsaw University of Technology, Warsaw, Poland; 13 Division of Oncology, Stanford University Medical Center, Stanford, CA USA

**Keywords:** Breast cancer, Brain metastasis, HER2, *RAD51*, *HDGF*, *TPR*

## Abstract

The overexpression or amplification of the human epidermal growth factor receptor 2 gene (*HER2*/*neu*) is associated with high risk of brain metastasis (BM). The identification of patients at highest immediate risk of BM could optimize screening and facilitate interventional trials. We performed gene expression analysis using complementary deoxyribonucleic acid-mediated annealing, selection, extension and ligation and real-time quantitative reverse transcription PCR (qRT-PCR) in primary tumor samples from two independent cohorts of advanced HER2 positive breast cancer patients. Additionally, we analyzed predictive relevance of clinicopathological factors in this series. Study group included discovery Cohort A (84 patients) and validation Cohort B (75 patients). The only independent variables associated with the development of early BM in both cohorts were the visceral location of first distant relapse [Cohort A: hazard ratio (HR) 7.4, 95 % CI 2.4–22.3; *p* < 0.001; Cohort B: HR 6.1, 95 % CI 1.5–25.6; *p* = 0.01] and the lack of trastuzumab administration in the metastatic setting (Cohort A: HR 5.0, 95 % CI 1.4–10.0; *p* = 0.009; Cohort B: HR 10.0, 95 % CI 2.0–100.0; *p* = 0.008). A profile including 13 genes was associated with early (≤36 months) symptomatic BM in the discovery cohort. This was refined by qRT-PCR to a 3-gene classifier (*RAD51*, *HDGF*, *TPR*) highly predictive of early BM (HR 5.3, 95 % CI 1.6–16.7; *p* = 0.005; multivariate analysis). However, predictive value of the classifier was not confirmed in the independent validation Cohort B. The presence of visceral metastases and the lack of trastuzumab administration in the metastatic setting apparently increase the likelihood of early BM in advanced HER2-positive breast cancer.

## Introduction

The overexpression or amplification of the human epidermal growth factor receptor 2 gene (*HER2*/*neu*) is associated with high risk of brain metastasis (BM). Approximately 30–50 % of advanced HER2-positive breast cancer patients will develop BM, with an annual risk of around 10 % [1–5]. It has been speculated that improvements in systemic therapy resulting in greater numbers and more durable systemic responses may permit more time for BM relapse. Trastuzumab, a monoclonal antibody that targets the extracellular domain of HER2, is used in combination with chemotherapy to improve the survival of patients with HER2-positive tumors [6–10]. However, owing to its high molecular weight, penetration of trastuzumab into the central nervous system is extremely low, 1/420th of serum levels [11], and this compound is ineffective in treating established BM.

The development of BM predictors in advanced breast cancer patients might have practical clinical implications. First, the use of imaging to detect occult BM in unselected patients is controversial, whereas this strategy may be reasonable in patients at highest immediate risk. Second, reliable predictive factors may improve selection of patients in clinical trials assessing the efficacy of putative BM prevention strategies, such as prophylactic cranial irradiation or the use of brain-permeable compounds. Finally, these studies may prompt new therapeutic strategies.

In the present study we analyzed the risk of early BM according to gene expression, and clinical and pathological variables in two well annotated cohorts of advanced HER2-positive breast cancer patients.

## Materials and methods

### Patients

**Fig. 1:**
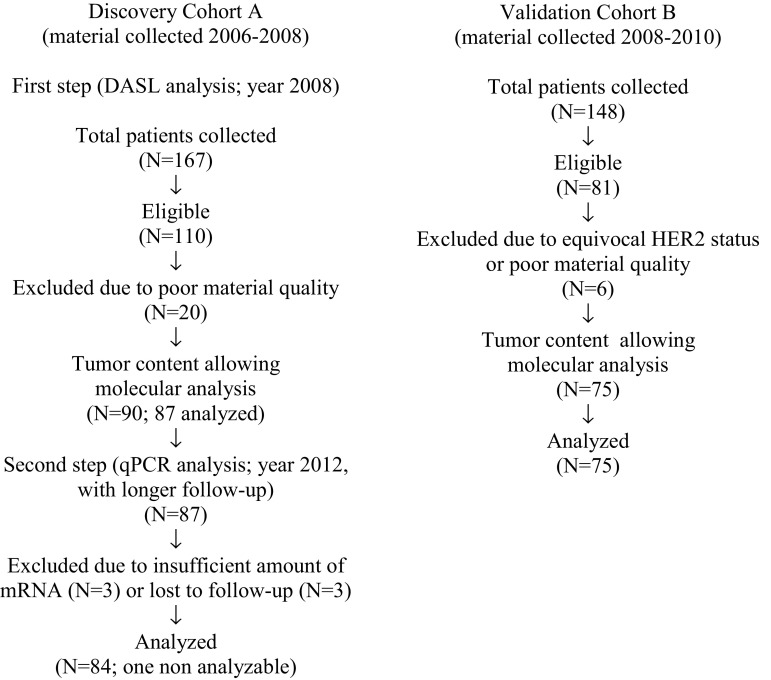
Consort diagram

This study was approved by the Institutional Review Board of the coordinating centers (Medical University of Gdańsk, Poland and Indiana University, USA). Two patient cohorts were derived from a consecutive series of 315 advanced HER2-positive breast cancer patients treated in nine oncology centers in Poland and Serbia between 1993 and 2010 (consort diagram; Fig. 1). Discovery Cohort A (*n* = 167) and an independent validation Cohort B (*n* = 148) were collected between 2006–2008 and 2008–2010, respectively. According to standard clinical practice, no screening for occult BMs was used, therefore almost all BM were symptomatic. BM were defined as metastatic lesions involving the brain parenchyma, with or without accompanying leptomeningeal disease. Demographic and clinicopathologic data, as well as treatments and clinical follow-up were extracted from institutional databases or original patient files. Treatments were rule based (Table 1). Dominant metastatic sites were assigned into three categories: soft tissue, bones and viscera. Dominant metastatic site was classified by the category associated with the worst prognosis in the following order of increasing gravidity: soft tissue, bones, viscera [12].

**Table 1:** Patient characteristics

Variables	Discovery Cohort A *N* = 84	Validation Cohort B *N* = 75	*p*
Age at diagnosis			
Mean	50	52	0.07
Range	29–75	28–71	
Age at brain metastases			
Mean	47.6	51.5	**0.03**
Range	30–64	33–69	

	*N*	%	*N*	%	
Brain metastases					
No	36	43	34	45	0.75
Yes	48	57	41	55	
Menopausal status					
Premenopausal	43	51	28	37	0.07
Postmenopausal	40	48	47	63	
Histology					
Ductal	70	83	58	77	**0.02**
Lobular	6	7	5	7	
Other	1	1	3	4	
Uncertain	0	0	7	9	
Ductal and lobular	6	7	2	3	
Grades					
2	35	46	34	49	0.69
3	41	54	35	51	
ER (IHC)					
Negative	52	63	43	57	0.49
Positive	31	37	32	43	
PR (IHC)					
Negative	59	71	54	72	0.89
Positive	24	29	21	28	
Breast cancer surgery					
No	16	19	15	20	0.91
Yes	67	81	60	80	
Radiotherapy					
No	38	46	25	34	0.12
Adjuvant	27	32	28	37	
Definitive	0	0	1	1	
Palliative	13	17	10	13	
Combination thereof	4	5	11	15	
Chemotherapy induction					
No	49	59	55	73	0.06
Yes	34	41	20	27	
Chemotherapy					
No	2	2	1	1	0.22
Adjuvant	11	13	19	25	
For metastatic disease	15	18	9	12	
Combination thereof	56	67	46	61	
Trastuzumab therapy					
No	13	16	12	16	0.72
Adjuvant	2	2	2	3	
For metastatic disease	68	81	58	77	
Combination thereof	1	1	3	4	
Anti-HER2 tyrosine kinase inhibitors					
No	72	86	51	68	**0.02**
Adjuvant	0	0	2	3	
For metastatic disease	12	14	22	29	
Endocrine therapy					
No	44	52	41	55	0.85
Adjuvant/neoadjuvant	21	25	21	28	
For metastatic disease	8	10	6	8	
Combination thereof	11	13	7	9	
Type of first progression					
Local	2	2	10	13	**0.02**
Regional	3	4	3	4	
Distant	77	92	56	75	
Local/regional and/or distant	2	2	6	8	
Dominant site of metastatic disease					
Soft tissue	4	5	16	22	**0.001**
Bone	3	4	7	10	
Visceral	77	91	50	68	
Location of first extracranial visceral relapse					
Lung	23	28	25	35	0.19
Liver	34	41	26	36	
Other	16	19	15	21	
Lung and liver	8	10	2	3	
Lung and other	0	0	3	4	
Liver and other	2	2	1	1	
Brain as first relapse					
No	77	93	67	89	0.45
Yes	6	7	8	11	

Significant values marked in bold

*ER* estrogen receptor, *PR* progesterone receptor, *HER2* human epidermal growth factor receptor 2

### Pathology review

The starting material from each patient was a formalin-fixed, paraffin embedded specimen of primary breast cancer. A pre-cut section of each tumor, stained with hematoxylin and eosin, was reviewed by two pathologists (SB and WB) to confirm the presence of sufficient invasive breast cancer component (1 cm^2^ invasive tissue, ≥30 % tumor cells). In Cohorts A and B, 90/167 and 75/148 tumors, respectively, had sufficient material for molecular analysis. Expression of ER and PR was determined using immunohistochemistry (IHC), with 10 % of nuclear staining considered as a positive result. HER2 protein expression was determined using semiquantitative IHC (HercepTest, Dako A/S, Glostrup, Denmark) or HER-2/neuTest 4B5 (Ventana Medical Systems, Inc.). Only samples showing strong expression (scored 3+), defined as uniform, and intense membrane staining of at least 10 % of invasive tumor cells, were considered positive. The samples showing intermediate expression (scored 2+) were subjected to additional analysis of *HER2* gene copy number using fluorescence in situ hybridization (FISH). Gene amplification by FISH was defined as a FISH ratio (HER2/centromeric probe for chromosome 17 ratio) of greater than 2.0. FISH-positive patients were considered HER2-positive.

### RNA extraction

Tumor cells were processed using macrodissection to enrich their population for analysis. Sections were deparaffinized with CitriSolv clearing agent (Fisher Scientific Company, Fair Lawn, NJ) and scraped off from the slide into a microcentrifuge tube. Total RNA was extracted from three 10 μm thick whole tissue sections from each sample using the Roche high pure RNA paraffin kit according to manufacturer’s instructions (Roche Applied Science, Indianapolis, IN). Purified total RNA samples were stored frozen at −80 °C until needed for quality control (QC) analysis and subsequent gene expression profiling and quantitative reverse transcription PCR (qRT-PCR). The concentration of RNA was measured using Nanodrop^®^ ND-1000 spectrophotometer (ThermoScientific, Wilmington, DE). RNA (200 ng) was reverse-transcribed to complementary deoxyribonucleic acid (cDNA) using iScript cDNA synthesis kit (Bio-Rad Laboratories, Inc., Hercules, CA). To prequalify RNA samples, SYBR Green-based qRT-PCR (Applied Biosystems, Foster City, CA) was performed for *RPL13*A ribosomal protein gene according to Illumina’s instructions (San Diego, CA).

### DASL analysis

Cohort A samples were analyzed by annealing, selection, extension and ligation (DASL) assay using Cancer Panel v1 to provide expression data on 502 known cancer genes. DASL was performed with the Sentrix universal array (Illumina, San Diego, California) as per the manufacturer’s instructions [13] and blinded to patient outcome. Shortly, a 20-μl RT reaction containing a reaction mix (MMC; Illumina, San Diego, CA), biotinylated random hexamers and oligo-d(T)_18_, and total RNA, was incubated at room temperature for 10 min and then at 42 °C for 1 h. Pooled assay oligos were annealed to their sequence-specific targets on the cDNA under a controlled hybridization program. The cDNA was immobilized on paramagnetic beads and washed to remove any excess or mis-hybridized oligos. Hybridized oligos were then extended and ligated to generate amplifiable templates, using Illumina-supplied reagents and conditions (BeadStation User’s Manual, Illumina). A PCR reaction was performed with Cy3 labeled universal PCR primers. Single-stranded PCR products were prepared by denaturation, and were then hybridized to Sentrix arrays under a temperature gradient program. The arrays were imaged using a BeadArray Reader scanner (Illumina). The DASL assay was performed three times independently, and samples were hybridized to three different array matrices. The 502-gene assay was available in a 96-well format; this enabled analysis of all the samples in a single batch. Built-in internal controls and replicate samples were used to analyze stability of the assay. The *r* ^2^ values for the duplicate samples were greater than 0.95.

### Generation of the 13-gene signature

Cohort A samples were divided into an internal training set and an internal testing set. Predictive analysis of microarray analysis (http://www-stat.stanford.edu/~tibs/PAM/) was performed to identify multigene profiles predictive for BM. The best gene-expression signature was selected based on a built-in 10-fold cross-validation analysis in PAM. Then the gene-signature was output as a single variable from the PAM. Its association with the BM free survival (BMFS) was analyzed in the internal testing set with a Cox regression analysis, in which clinical and demographic variable effects were justified. This analysis was performed with the R function, coxph. The gene signature construction from the internal training set used the optimal variable selection strategy in PAM, and *p* value was not considered. Then, the correlation between the gene signature and BMFS was assessed by the Cox regression model, and the *p* value <0.05 was considered as statistically significant.

### Real-time qRT-PCR analysis

Owing to the abandoning of the 502-gene DASL assay by the manufacturer, and to increase the potential utility of the profile, we switched to a qRT-PCR assay. Apart from its clinical applicability, this method allows precise quantification of transcriptional abundance of identified genes. TaqMan reactions were performed in triplicates using custom array microfluidic cards preloaded with TaqMan gene expression assays containing 16 genes (13 discriminant genes and 3 reference genes) on an ABI Prism 7900HT fast real-time platform according to the manufacturer’s instructions. The primer sequences are listed in Table 2. Transferrin receptor (*TFRC*), beta cytoskeletal actin (*ACTB*) and glyceraldehyde-3-phosphate dehydrogenase (*GAPDH*) were used as endogenous reference controls for normalization. Delta threshold cycle (ΔC_t_) values for each of the 13 genes of interest were normalized using the three endogenous reference controls according to the method of Applied Biosystem’s DataAssist™ Software. All procedures were performed blinded to patient outcomes. After normalization, $2^{{ - \Updelta {\text{C}}_{\text{t}} }}$ values were subject to the leave-one-out cross-validated linear discriminant analysis (LDA), and coefficients for the individual genes were chosen. The coefficients for the individual genes and individual gene expression data for each patient were collated to develop an individual score, which was used for statistical analysis in both cohorts.

**Table 2:** List of genes constituting a 13-gene profile and TaqMan probes used in qRT-PCR analysis

Gene symbols	Gene names	Human assay ID	Amplicon length (bp)
*CDK4*	Cyclin dependent kinase 4	Hs00175935_m1	65
*CCNC*	Cyclin C	Hs00193177_m1	78
*PTK2*	Focal adhesion kinase (protein tyrosine kinase 2)	Hs00178587_m1	68
*MYC*	v-myc avian myelocytomatosis viral oncogene homolog	Hs99999003_m1	65
*BARD1*	*BRCA1* associated RING domain 1	Hs00184427_m1	73
*RAD51*	RAD51 homolog	Hs00153418_m1	58
*FANCG*	Fanconi anemia group G	Hs00184947_m1	116
*PCNA*	Proliferating cell nuclear antigen	Hs00696862_m1	95
*PRCC*	Papillary renal cell carcinoma-translocation associated	Hs00410541_m1	77
*TPR*	Translocated promoter region	Hs00162918_m1	82
*CTTN*	Cortactin	Hs00193322_m1	81
*DSP*	Desmoplakin	Hs00189422_m1	74
*HDGF*	Hepatoma-derived growth factor	Hs00610314_m1	110
*ACTB*	Actin, beta, cytoplasmic	Hs00357333_g1	77
*GAPDH*	Glyceraldehyde-3-phosphate dehydrogenase	Hs99999905_m1	122
*TFRC*	Transferrin receptor	Hs99999911_m1	105

Selected controls: *ACTB*, *GAPDH*, *TFRC*

### Statistical analysis

All statistical analyses were performed using STATA software version 11. Statistical significance was defined as *p* < 0.05. Gene expression data were normalized at the median level. Hierarchical clustering and singular value decomposition methods were applied to detect the outliers for QC purposes. The false discovery rate (FDR), an estimate of the proportion of errors committed by falsely rejecting null hypotheses was calculated for each gene. Categorical variables in both cohorts (including correlation of the developed 3-gene classifier with clinicopathologic variables) were compared using Pearson’s Chi squared test (*χ* ^2^). Survival curves were plotted using Kaplan–Meier method starting from date of primary breast cancer diagnosis to date of death or last follow-up. The BMFS was a primary endpoint and was defined from date of primary breast cancer diagnosis to date of BM diagnosis, death of any cause, or date of last follow-up. Univariate survival analysis and time to diagnosis of BM within 36 months in Cohorts A and B were performed with log-rank test, Wilcoxon test and Cox proportional hazard regression and controlled for the competing risk of death [14]. Multivariate analysis used a stepwise forward selection of univariate model with *p* ≤ 0.20.

## Results

### Characteristics and outcomes of study cohorts

#### Discovery Cohort A

Of the 84 primary tumors subjected to analysis in the Cohort A, 83 were analyzable (Fig. 1). The patient mean age was 48 years (range 30–64), with the patients roughly divided between pre- and post-menopausal status (Table 1). Eighty three percent of the tumors were invasive ductal cancers, 63 % were ER-negative and 71 % PR-negative. Ninety two percent of patients had dominant visceral metastatic disease; 98 % of patients received chemotherapy and 48 % endocrine therapy in adjuvant and/or metastatic setting. More than 40 % of patients received induction chemotherapy and 87 % of patients were administered trastuzumab in adjuvant or metastatic setting, usually in combination with chemotherapy. In 14 % of patients lapatinib was administered at trastuzumab relapse. Follow-up from breast cancer diagnosis varied from 1 to 185 months. Within this period, 48 patients developed symptomatic BM. The median time from initial breast cancer to BM diagnosis was 36 months (range 2–141 months). In 7 % of patients brain was the first site of distant relapse, with or without accompanying extracranial relapse. After BM, HER2 directed treatments included trastuzumab (33 % of patients), lapatinib (15 %) and either used sequentially (4 %). Seventy seven percent of patients received cranial radiotherapy. The median overall survival (OS) from the initial diagnosis of breast cancer was 44 months (range 0.9–185 months).

#### Validation Cohort B

The Cohort B, including 75 analyzable cases, was similar to Cohort A in terms of age, ER and PR expression, and tumor grade (Table 1). However, Cohort B included significantly more non-ductal cancers, patients were older at BM development and had different patterns of relapse (fewer distant relapses as the first failure, more first relapses in the visceral organs and fewer visceral dominant metastatic sites). Furthermore, more patients in this cohort received lapatinib following failure of trastuzumab. The median time to diagnosis of BM in this cohort was longer (40 months; range 0.33–125 months, compared to 36 months in Cohort A), and so was the median OS (50 months; range 11–186 months, compared to 44 months in Cohort A). In Cohort B 41 patients developed BM, including 16 that occurred within 36 months from diagnosis.

### Determinants of BMFS and OS

Performed in Cohort A binary comparison for presence or absence of BM among 502 analyzed genes did not show any differential gene expression (25 having *p* < 0.05, a FDR of 1.0). However, a gene expression analysis in 22 and 21 patients who developed BM within 36 months (the median time to diagnosis of BM) versus thereafter, respectively, identified differential expression of 48 genes with *p* < 0.01 and FDR = 0.1. Predictive analysis of microarray analysis identified a 13-gene profile [cyclin dependent kinase 4 (*CDK4*), cyclin C (*CCNC*), focal adhesion kinase (protein tyrosine kinase 2, *PTK2*), v-myc avian myelocytomatosis viral oncogene homolog (*MYC*), BRCA1 associated RING domain 1 (*BARD1*), *RAD51* homolog (*RAD51*), Fanconi anemia group G (*FANCG*), proliferating cell nuclear antigen (*PCNA*), papillary renal cell carcinoma-translocation associated (*PRCC*), translocated promoter region (*TPR*), cortactin (CTTN), desmoplakin (*DSP*), hepatoma-derived growth factor (*HDGF*)] at effectively distinguished patients with early versus late BM [hazard ratio (HR) 5.6, 95 % CI 1.9–16.5; *p* = 0.002 in the univariate analysis; HR 8.5, 95 % CI 2.6–28.0; *p* < 0.001 in the multivariate analysis; Table 3]. The microarray data have been deposited in NCBI’s gene expression omnibus (http://www.ncbi.nlm.nih.gov/geo; GSE38057).

**Table 3:** Factors associated with early brain metastases (≤36 months)

Variables	Univariate analysis			Multivariate analysis*		
	HR	95 % CI	*p*	HR	95 % CI	*p*
Discovery Cohort A						
ER negative versus positive	3.3	1.1–10.0	**0.03**	2.8	0.9–9.1	0.07
Visceral site of first distant relapse	4.5	1.9–10.7	**0.001**	7.4	2.4–22.3	**<0.001**
13-Gene expression high versus low	5.6	1.9–16.5	**0.002**	8.5	2.6–28.0	**<0.001**
3-Gene classifier high versus low	3.7	1.3–11.1	**0.01**	5.3	1.6–16.7	**0.005**
Trastuzumab therapy for metastatic disease no versus yes	3.3	1.1–10.0	**0.02**	5.0	1.4–10.0	**0.009**
Validation Cohort B						
ER negative versus positive	2.5	0.9–10.0	0.09	5.0	1.1–10.0	**0.04**
Visceral site of first distant relapse	5.9	1.8–19.7	**0.003**	6.1	1.5–25.6	**0.01**
3-Gene classifier high versus low	1.2	0.3–20.0	0.8	NC		
Grade high versus low	3.3	1.1–14.3	**0.03**	3.8	0.9–16.7	0.07
Trastuzumab therapy for metastatic disease no versus yes	2.5	1.0–10.0	0.06	10.0	2.0–100.0	**0.008**

**Fig. 2:**
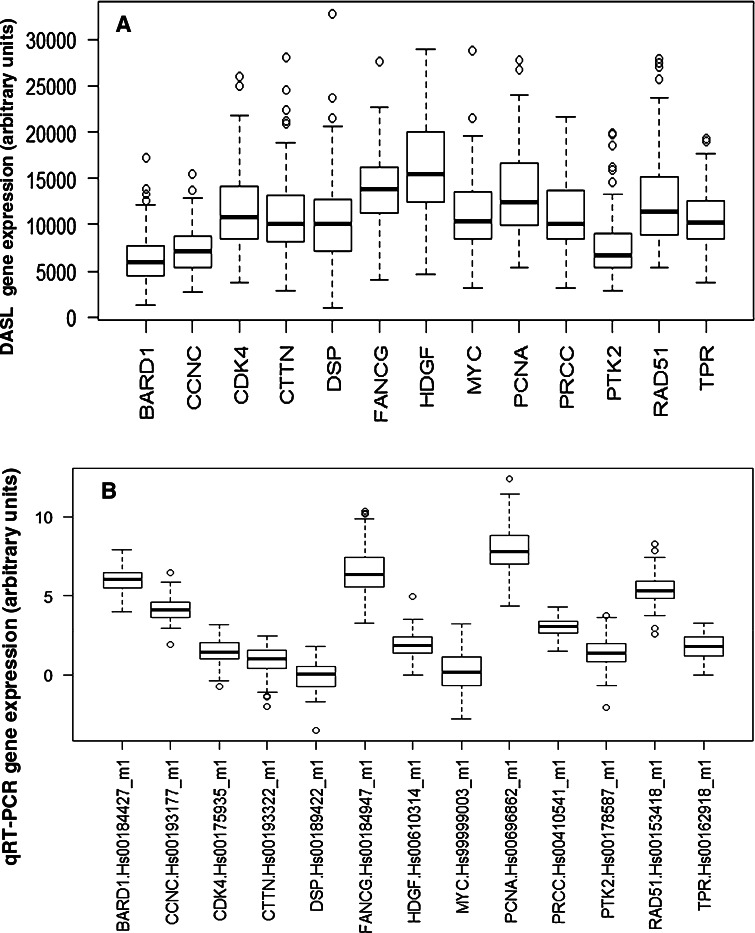
Cohort A. Distribution of the 13 gene transcript expression obtained from the RNA isolation process in DASL and qRT-PCR. **a** DASL (data was normalized using quantile normalization), **b** qRT-PCR normalized using the endogenous reference controls (*ACTB*, *GAPDH*, *TFRC*). The graph shows apparent inter-panel discordance of *BARD1*, *CCNC* and *HDGF* expression, and minor inter-panel discordance of *FANCG* and *PCNA* expression

In order to increase the potential clinical applicability of this signature, a qRT-PCR based analysis of the 13 genes (and 3 references) was performed and showed promising preliminary results [15, 16]. The TaqMan gene expression assay IDs for each gene was chosen to meet FFPE sample requirements for custom TLDA based on Applied Biosystems guidelines. As expected, DASL and qRT-PCR had inherent differences related to the platform (Fig. 2). As the next step, a leave-one-out LDA was performed using an updated database that had a longer follow-up (5 years) data. A predictive model that included only 3 of the original 13 genes: *HDGF*, *RAD51* and *TPR*, with corresponding LDA coefficients of 1.06, 0.35 and −1.08, respectively, was developed. The 3-gene classifier was highly predictive of early BM both in univariate (HR 3.7, 95 % CI 1.3–11.1; *p* = 0.01) and multivariate analysis (HR 5.3, 95 % CI 1.6–16.7; *p* = 0.005; Table 3). High 3-gene classifier was associated with tumor grade 3, ER-negativity and less frequent use of endocrine treatment and trastuzumab in the adjuvant and/or metastatic setting (Table 4). Additionally, patients with high 3-gene classifier were more likely to develop the first relapse in the visceral organs.

**Table 4:** Relationship between the 3-gene classifier and other variables

Variables	3-Gene classifier									
	Discovery Cohort A (*N* = 83)					Validation Cohort B (*N* = 75)				
	Low (*N* = 34; 41 %)		High (*N* = 49; 59 %)			Low (*N* = 63; 84 %)		High (*N* = 12; 16 %)		
	*N*	%	*N*	%	*p*	*N*	%	*N*	%	*p*
Menopausal status										
Premenopausal	20	60	23	47		24	38	4	33	0.75
Postmenopausal	13	40	26	53	0.22	39	62	8	67	
Primary tumor grades										
2	20	67	15	33		28	49	6	50	0.95
3	10	33	30	67	**0.05**	29	51	6	50	
ER										
Negative	16	47	36	75		35	56	8	67	0.47
Positive	18	53	12	25	**0.01**	28	44	4	33	
PR										
Negative	23	68	36	75		45	71	9	75	0.80
Positive	11	32	12	25	0.46	18	29	3	25	
Induction chemotherapy										
No	21	62	28	58		50	79	5	42	**0.007**
Yes	13	38	20	42	0.75	13	21	7	58	
Endocrine therapy										
No	12	36	31	63		33	52	8	67	0.36
Yes	22	65	18	37	**0.01**	30	48	4	33	
Trastuzumab for metastatic disease										
No	3	9	13	26		15	24	2	17	0.59
Yes	31	91	36	73	**0.04**	48	76	10	83	
Visceral location of first relapse										
No	31	94	34	69		44	73	9	75	0.90
Yes	2	6	15	31	**0.01**	16	27	3	25	
First site of visceral metastasis										
Lung	11	33	12	25		22	37	3	25	**0.05**
Liver	16	48	18	37		22	37	4	33	
Other	2	6	13	27		13	22	2	17	
Lung and liver	4	12	4	8		0	0	2	17	
Lung and other	0	0	0	0		2	3	1	8	
Liver and other	0	0	2	4	0.19	1	2	0	0	
Brain metastases										
No	11	32	25	51		28	44	6	50	
Yes	23	67	24	49	0.09	35	55	6	50	0.72

Significant values marked in bold

*ER* estrogen receptor, *PR* progesterone receptor, *HER2* human epidermal growth factor receptor 2

In an independent Cohort B the mean qRT-PCR expression of 13 genes was different compared to Cohort A, and only 16 % of patients (compared to 59 % in Cohort A) were assigned to the high-risk group (Table 4). Accordingly, the 3-gene classifier was not predictive of early BM (HR 1.2, 95 % CI 0.3–20.0; *p* = 0.8; Table 3). In this cohort the high 3-gene classifier was associated with less frequent use of induction chemotherapy and more lung and liver metastases (Table 4).

In the multivariate analysis, in both cohorts the visceral location of first distant relapse (Cohort A: HR 7.4, 95 % CI 2.4–22.3; *p* < 0.001; Cohort B: HR 6.1, 95 % CI 1.5–25.6; *p* = 0.01) and the lack of trastuzumab administration in the metastatic setting (HR 5.0, 95 % CI 1.4–10.0; *p* = 0.009 and HR 10.0, 95 % CI 2.0–100.0; *p* = 0.008, respectively) correlated with early BM (Table 3). ER-negativity had a strong trend in Cohort A (HR 2.8, 95 % CI 0.9–9.1; *p* = 0.07) and was significant in Cohort B (HR 5.0, 95 % CI 1.1–10.0; *p* = 0.04).

In both cohorts the independent variables associated with shorter OS included higher tumor grade (HR 1.9, 95 % CI 1.1–3.3; *p* = 0.02; HR 1.9, 95 % CI 1.4–3.2; *p* = 0.03), ER negativity (HR 2.0, 95 % CI 1.1–3.3; *p* = 0.03; HR 2.5, 95 % CI 1.4–5.0; *p* < 0.01) and the lack of primary tumor surgery (HR 5.0, 95 % CI 2.0–10.0; *p* < 0.01; HR 3.3, 95 % CI 1.7–10.0; *p* < 0.01).

## Discussion

The aim of this study was to identify molecular predictors of the BM development in advanced HER2-positive breast cancer patients. This subset of breast cancer patients carry particularly high risk of BM. Additionally, some studies suggested increased risk of BM associated with the use of trastuzumab [17].

The current study employed a high throughput DASL technology based on the expression of 502 cancer related genes in addition to analysis of the clinicopathologic variables. This targeted gene analysis did not demonstrate any differential gene expression in patients who did and did not develop BM. This may likely be due to the limited number of genes analyzed, but it is also possible that BM in advanced HER2-positive breast cancer patients is a biologically determined, stochastic and inevitable event. Further analysis of the DASL led to identification of a 13-gene profile that was apparently predictive for development of early BM [15]. For precise quantification of transcriptional abundance of identified genes, we employed qRT-PCR technology, which identified a 3-gene classifier (*RAD51*, *HDGF*, *TPR*), also seemingly predictive for early BM. However, the significance of this classifier was not confirmed in the independent cohort.

The retrospective design of this study made it difficult to control for major clinicopathologic differences between Cohorts A and B. In consequence, patients in Cohort B had fewer ductal carcinomas and, even more importantly, less frequently received neoadjuvant chemotherapy. Gene expression alterations of breast cancer were recently demonstrated to be drug-specific, and drug-induced tumor gene signatures may be more informative than unchallenged signatures in predicting treatment outcomes [18, 19]. The study by Bos et al. [20] showed that BM gene set tested in various breast cancer cohorts was less BM predictive in patients whom received postoperative systemic therapy compared to those whom did not. This confirms the hypothesis that systemic therapies, apart from their preventive effect, may also alter the pattern of relapse in breast cancer. In this study, patients in Cohort B, compared to Cohort A, had also infrequent first relapse at distant sites and significantly fewer visceral metastases. Furthermore, much more patients in this cohort received lapatinib at trastuzumab relapse (32 %, compared to 14 % in Cohort A). The pivotal study by Geyer et al. [21] showed that the addition of lapatinib to capecitabine after progression on trastuzumab resulted in decreased BM occurrence, and preclinical studies show that lapatinib prevents BMs formation by 53 % in a HER2-transfected model system [22]. The abovementioned differences between both cohorts led to better general prognosis in Cohort B compared to Cohort A, expressed by longer OS and time to diagnosis of BM. Finally, the imbalanced proportion of patients with high gene classifier in both cohorts (59 % in Cohort A vs. 16 % in Cohort B) might have largely impacted study results.

Although the gene signature could not be validated, it identified a number of genes that could be important in the development of BM. The most important of which is *RAD51*, a gene involved in homologous recombination in DNA double strand breaks repair [20]. *RAD51* expression has been linked to response to neoadjuvant therapy [23–25]. We have previously reported that high cytoplasmic expression of RAD51 in breast cancer is associated with significantly increased risk of BM, particularly in combination with high Ki-67 index and ER-negativity [26]. Further, in other study demonstrated that BARD1 and RAD51 are frequently overexpressed in BMs from breast cancer and may constitute a mechanism to overcome reactive oxygen species-mediated genotoxic stress in the metastatic brain [27]. Taken together, this data suggest that RAD51 targeting might be important in HER2-positive breast cancer. High nuclear expression of *HDGF*, another gene constituting our 3-gene signature, was earlier found to associate with high tumor grade, Ki-67 >20 %, lymph node involvement and poor prognosis in breast cancer patients [28, 29]. Chen et al. [29] demonstrated that nuclear HDGF over-expression stimulates epithelial–mesenchymal transition of breast cancer cells by down-regulation of E-cadherin and up-regulation of vimentin. The third gene of our signature—*TPR*, a translocated promoter region nuclear basket protein, is poorly characterized but has a normal function in nuclear pore function and is the target of oncogenic fusions [30].

In the current study, the clinical factors associated with early development of BM were visceral location of first relapse and, at a borderline level, ER-negativity, the two hallmarks of tumor aggressiveness. This is partly consistent with our earlier study in advanced HER2-positive breast cancer patients, showing the association between the risk of BM and shorter time to first extracranial progression [5]. The association between ER-negativity and the occurrence of BM in HER2-positive breast cancer patients was earlier reported by other authors [2, 4, 31, 32]. Indeed, the clinical behavior including tumor kinetics and sites of recurrence in ER-positive/HER2 positive (HER2-positive luminal B) breast cancer is different compared to that in non-luminal HER2 enriched subtype [31–34]. We also showed that trastuzumab administration in the metastatic setting may reduce the risk of early BM. This is in line with two other studies, that noticed shorter time to development of BM in HER2-positive patients who never received trastuzumab [35, 36].

## Conclusions

We demonstrated that the presence of visceral metastases and the lack of trastuzumab administration in the metastatic setting apparently increase the likelihood of early BM in advanced HER2-positive breast cancer, and the 3-gene classifier does not improve their predictive value. Our study also illustrates the difficulties in developing clinically useful predictive markers in the retrospective setting [37]. In our case these included problems associated with archival tissue collection, heterogeneity of patient populations and inconsistent therapeutic approaches over the study period. Further studies, including larger and more homogeneous groups, are necessary to identify biomarkers, which may help in designing BM preventive trials and prompt new treatment strategies.
